# AutoML-ID: automated machine learning model for intrusion detection using wireless sensor network

**DOI:** 10.1038/s41598-022-13061-z

**Published:** 2022-05-31

**Authors:** Abhilash Singh, J. Amutha, Jaiprakash Nagar, Sandeep Sharma, Cheng-Chi Lee

**Affiliations:** 1 Indian Institute of Science Education and Research Bhopal, Fluvial Geomorphology and Remote Sensing Laboratory, Bhopal, 462066 India; 2 Gautam Buddha University, School of ICT, Greater Noida, 201312 India; 3 Indian Institute of Technology Kharagpur, Subir Chowdhury School of Quality and Reliability, Kharagpur, 721302 India; 4 Department of Electronics Engineering, Madhav Institute of Technology and Science, Gwalior, 474005 India; 5 Department of Library and Information Science, Research and Development, Center for Physical Education, Health, and Information Technology, Fu Jen Catholic University, New Taipei, 242 Taiwan; 6 Department of Computer Science and Information Engineering, Asia University, Taichung, 41354 Taiwan

**Keywords:** Energy science and technology, Mathematics and computing, Physics

## Abstract

Momentous increase in the popularity of explainable machine learning models coupled with the dramatic increase in the use of synthetic data facilitates us to develop a cost-efficient machine learning model for fast intrusion detection and prevention at frontier areas using Wireless Sensor Networks (WSNs). The performance of any explainable machine learning model is driven by its hyperparameters. Several approaches have been developed and implemented successfully for optimising or tuning these hyperparameters for skillful predictions. However, the major drawback of these techniques, including the manual selection of the optimal hyperparameters, is that they depend highly on the problem and demand application-specific expertise. In this paper, we introduced Automated Machine Learning (AutoML) model to automatically select the machine learning model (among support vector regression, Gaussian process regression, binary decision tree, bagging ensemble learning, boosting ensemble learning, kernel regression, and linear regression model) and to automate the hyperparameters optimisation for accurate prediction of numbers of *k*-barriers for fast intrusion detection and prevention using Bayesian optimisation. To do so, we extracted four synthetic predictors, namely, area of the region, sensing range of the sensor, transmission range of the sensor, and the number of sensors using Monte Carlo simulation. We used 80% of the datasets to train the models and the remaining 20% for testing the performance of the trained model. We found that the Gaussian process regression performs prodigiously and outperforms all the other considered explainable machine learning models with correlation coefficient (R = 1), root mean square error (RMSE = 0.007), and bias = − 0.006. Further, we also tested the AutoML performance on a publicly available intrusion dataset, and we observed a similar performance. This study will help the researchers accurately predict the required number of *k*-barriers for fast intrusion detection and prevention.

## Introduction

Intrusion detection at border areas is of utmost importance and demands a high level of accuracy. Any failure in intrusion detection may result in havoc on the nation’s security^1^. Each country shares international boundaries with its neighboring countries, extending to thousands of kilometers. Continuous monitoring of such a colossal borderline through occasional patrolling is a crucial problem. To overcome this problem, WSNs are generally used and deployed along the borderline for surveillance and monitoring^2,3^. WSNs are a widely adopted technology that consists of a group of sensors capable of sensing, processing, and transmitting processed information. It can be easily installed anywhere, even in hard-to-reach areas, because it does not require pre-installed infrastructure. The capability of detecting any event or environmental condition makes it more prudent for intrusion detection applications^4,5^. Apart from intrusion detection, WSNs found applications in precision agriculture, health monitoring, environment monitoring, hazards monitoring, and many more^6–9^.

Border surveillance, intrusion detection, and prevention problems are addressed with two different approaches. Researchers propose various algorithms and Internet of Things (IoT) solutions for intrusion detection and surveillance in border areas in the first approach. In the second approach, they develop analytical models to estimate the intrusion detection probability in terms of *k*-coverage, *k*-barrier coverage, number of *k*-barriers, and many other performance metrics. Yang et al.^10^ have proposed an energy-efficient intrusion detection method that is capable of identifying weak zones of the network deployment region that need to be repaired. After identifying the weak zones, they are repaired to achieve the desired quality of barrier coverage. Specifically, their proposed method focuses on one-directional coverage only for single and multiple intruder scenarios. The authors have claimed that their proposed method and algorithms could enhance the network lifetime. In another work presented in^11^, Raza et al. have analysed the impact of heterogeneous WSNs deployed following either uniform or Gaussian distribution scenario. They have studied the impact of sensor density and sensing range of sensor nodes on the intrusion detection probability. They found that the heterogeneous WSNs provide better intrusion detection performance than the homogeneous WSNs at a given sensing range and sensor node density. Similarly, Arfaoui et al.^12^ have rendered an analytical model that considers the notion of possible paths that an intruder can follow to cross a belt region in border areas. They have developed a model considering border area characteristics and the intrusion paths to estimate the time taken by an intruder to cross the border area. The authors conclude that their proposed model can detect the intrusion as soon as an intruder enters the restricted border area.

Further, Singh and Singh^13^ have presented a smart border surveillance system that uses a WSN which is able to identify and detect the intrusion and then alerts the control center about the presence of an intruder. The proposed system is capable in differentiating between animals and persons. Further, the system uses Raspberry Pi boards integrated with infra-red, ultrasonic and camera sensors and is found to be very effective and accurate to identify any possible intruder. Again, Sharma and Kumar^14^ have proposed a ML-based smart surveillance and intrusion detection system for border regions. The proposed system is capable in detection intruders during day time and at night along with the kind of weapon carried by the intruder. The proposed system is made of a high-resolution camera with IR capabilities for day and night vision, a GPS module interfaced with Raspberry Pi to extract the accurate location of the intruder, and a bluetooth scanner to detect the bluetooth signature of the intruder device. The entire module is put into a climate protected box that can be mounted on a high platform. Further, Mishra et al. in^15^ have provided a detailed literature review on various ML techniques for intrusion detection. They have also provided a comprehensive discussion on various types of attacks along with their respective features and security threats. With the help of a specific feature, ML techniques can identify and detect the intrusion quickly and accurately. Sun et al.^16^ have proposed a three-level intrusion detection model to minimise the memory consumption, computational time, and cost. The proposed model is claimed to decrease memory consumption, time, and cost up to a great extend. Further, in^17^, Ghosh et al. have proposed two routing schemes, namely KPS and Loop-Free (LP)-KPS, to enhance the lifetime of a WSN deployed for intrusion detection in border areas or surveillance of some crucial military establishments. On comparing the proposed algorithms with LEACH and TEEN routing algorithms, they found that the proposed algorithms provide enhanced network lifetime. In^18^, Benahmed and Benahmed have proposed an optimal approach to achieve a fault-tolerant network for the surveillance of critical areas using WSNs. The proposed approach identifies the faulty sensors and replaces them with active sensors to fill the coverage gap. The proposed approach can provide a sufficient minimum number of sensors to cover the area under surveillance. Another work presented by Arfaoui and Boudriga in^19^ provided an efficient surveillance system that can rapidly detect any intruder crossing border areas. In this work, the authors have incorporated the impact of obstacles present in the environment and the terrain of the border areas to derive the expression for intrusion detection probability.

Further, Sharma and Nagar^20^ have obtained an analytical expression of *k*-barrier coverage probability for intrusion detection in a rectangular belt region. They have considered all the possible paths an intruder may follow to cross the region. Further, they have also analysed the impact of various parameters such as the number of sensors, sensing range, sensor to intruder velocity ratio, and the intrusion path angle.

The analytical approaches discussed above effectively solve the intrusion detection problem. However, these approaches need validation through the simulation approach, which is time-consuming. For example, a single iteration requires approximately 15 hours for a particular set of network parameters, increasing significantly as the network complexity increases. Various machine learning methods have been proposed to overcome the time-complexity issue associated with the simulations. Recently, Singh et al.^21^ proposed three machine learning methods based on GPR to map the *k*-barrier coverage probability for accurate and fast intrusion detection using WSNs. These methods are based on scaling the predictors; scale-GPR (S-GPR), center-mean-GPR (C-GPR), and GPR. They have used synthetic predictors derived from Monte Carlo simulations. They selected many sensors, sensing range of the sensor, sensor to intruder velocity ratio, mobile to static node ratio, angle of the intrusion path, and the required *k*-barriers as potential predictors. They found that the non-standardise methods accurately map the *k*-barrier coverage probability using the synthetic variables with R = 0.85 and RMSE = 0.095. More recently, Singh et al.^22^ proposed a logarithmic predictor transformation and scaling-based algorithm coupled with SVR (i.e., LT-FS-ID) to map the number of required *k*-barriers for fast intrusion detection and prevention over a rectangular Region of Interest (RoI) considering uniform sensor distribution. The dimension of the dataset LT-FS-ID is 182 $\times$ 5. They used four predictors to accurately predict the required *k*-barriers. They reported that the proposed approach accurately predicts the *k*-barriers with R = 0.98 and RMSE = 6.47. The feasibility of deep learning algorithms for the intrusion detection has been investigated by Otoum et al. in^23^. They have presented a restricted Boltzmann machine-based clustered IDS (RBC-IDS) for monitoring critical infrastructures using WSNs. Further, they have compared the performance of RBC-IDS with the adaptively supervised and clustered hybrid IDS (ASCH-IDS) and found that both provides same detection and accuracy rates, but, detection time of RBC-IDS is approximately twice that of ASCH-IDS.

The machine learning methods discussed above involve manual selection of the best performing algorithm, which may lead to bias results if the results are not compared with the benchmark algorithm. In addition, the optimisation of the hyperparameter associated with each algorithm is treated differently. To solve this problem, in this paper, we introduced an automated machine learning (AutoML) model to automate the model selection and hyperparameter optimisation task. In doing so, we synthetically extracted potential predictors (i.e., area of the region, sensing range of the sensor, transmission range of the sensor, and the number of sensors) through Monte Carlo simulation. We then evaluated the predictor importance and predictor sensitivity through the regression tree ensemble approach. Subsequently, we applied AutoML on the training datasets to get the best optimised model. We evaluated the performance of the best performing algorithm over the testing data using R, RMSE, and bias as performance metrics.

## Material and methods

### Predictor generation

The quality of the prediction of a machine learning model depends on the quality of predictors and the model hyperparameters^24^. These predictors can be categorised into real and synthetic-based upon the dataset acquiring process. The real data can be obtained through direct measurements through instruments or sensors. However, the generation of real data involves intensive cost and labor. In contrast to real data, synthetic data can be obtained through mathematical rules, statistical models, and simulations^25^. In comparison to real data, acquiring synthetic data is efficient and cost-effective. Due to this, the use of synthetic datasets to train machine learning models is increased in the past lustrum^21,26–29^.

We adopted the synthetic method to extract the predictor datasets using Monte Carlo simulations. In doing so, we have used network simulator NS-2.35 to generate the entire dataset. A finite number of homogeneous (i.e., sensing, transmission, and computational capabilities are identical for each sensor) sensor nodes are deployed according to Gaussian distribution, also known as a normal distribution in a rectangular RoI to achieve this. Gaussian distribution is considered in this study since it can improve intrusion detection capability and is preferred for realistic applications. In a Gaussian distributed network, the probability that a sensor node is located at a point *(x, y)* in reference to the deployed location (*x*$_{0}$, *y*$_{0}$)^30,31^ is given by:

1 $$\begin{aligned} f(x, y) = \frac{1}{2\pi \sigma _x\sigma _y}e^{-\left( \frac{(x-x_0)^2}{2\sigma _x^2} + \frac{(y-y_0)^2}{2\sigma _y^2}\right) } \end{aligned}$$

where $\sigma _x$ and $\sigma _y$ are the standard deviations of *x* and *y* location coordinates, respectively.

To evaluate the performance of WSNs, we have considered the Binary Sensing Model (BSM)^32^, which is the most extensively used sensing range model. Each sensor (*S* $_{i}$) is assumed with the sensing range (*R* $_{s}$) and is deployed at an arbitrary point (*P*(*x*$_{i}$, *y*$_{i}$)). As per BSM, the target can be detected by any random sensor with 100% probability if the target lies with in the sensing range of the sensor. Otherwise, the target detection probability will be equal to zero and is represented mathematically as:

2 $$\begin{aligned} P(S_{i}) = {\left\{ \begin{array}{ll} 1, \; if \; d(S_{i}, P) \le R_{s} \\ 0, \; otherwise \end{array}\right. } \end{aligned}$$

where ${d(S_{i}, P)} = \sqrt{(x_{i}-x)^{2} + (y_{i}-y)^{2}}$, the Euclidean distance between *S* $_{i}$ and target point *P*. In addition, we have considered that any two sensors can communicate if they satisfy the criteria, *R* $_{tx}$ $\ge$ 2*R*$_{s}$, where *R*$_{tx}$ and *R*$_{s}$ represents the transmission range and sensing range, respectively. A barrier is constructed by joining a cluster of sensor nodes across the RoI to detect the presence of intruders. Furthermore, to assure barrier coverage, it is required to identify a Barrier Path (BP) in the RoI. The sensor nodes detect each intruder in the path in this scenario. Thus, to ensure guaranteed *k*-barrier coverage in the rectangular RoI, the number of required nodes is computed as : *k* $= \lceil \frac{L}{2R_s}\rceil$ and maximum number of BPs can be computed as *BP* $_{max}$=$\lfloor \frac{N}{k}\rfloor$^33^, where *L* is the length of the rectangular RoI, * R*$_{s}$ is the sensing range of nodes, and *N* is the number of sensor nodes. Table 1 lists the various network parameters and their values that have been used to obtain the simulation results.

**Table 1:** Simulation parameters.

Parameters	Values
Network simulator	NS-2.35
Network region	Rectangular RoI
Network area ($\mathrm {m^2}$)	100 $\times$ 50–250 $\times$ 200
Sensor nodes (N)	100–400
Sensing range (R$_{\hbox {s}}$)	15–40 $\mathrm {m}$
Transmission range (R$_{\hbox {tx}}$)	30–80 $\mathrm {m}$
Node distribution	Gaussian distribution
Sensing model	Binary sensing model (BSM)

### Relative predictor importance

In machine learning, the choice of input predictors has a substantial control on its performance^28^. Predictor importance analysis is not restricted to any particular representations, techniques, or measures and can be used in any situation where predictive models are required. It is used to express how significant the predictor was for the model’s predictive performance, irrespective of the structure (linear or nonlinear) or the direction of the predictor effect. We calculated the relevancy of the selected predictors in estimating the *k*-barriers by estimating each predictor’s relative predictor importance score. To do so, we have used the regression tree ensemble technique^21,34^. It is an inbuilt class with a tree-based classifier that assigns a relative score for every predictor or attribute of the data. The higher the score, the more important the predictor.

Initially, we trained a regression tree ensemble model by boosting hundred regression trees (i.e., *t * = 100) with a learning rate of one (i.e., $\delta$ = 1) each using the Least Squares gradient Boosting (LSBoost) ensemble aggregation method. Boosting an ensemble of regression algorithms seems to have several advantages, like, handling missing data, representing nonlinear patterns, and yielding better generalisation if weak learners were combined into a single meta learner. In addition, the LSBoost ensemble minimises the mean square error by combining individual regression trees, often known as weak learners. The LSBoost technique successfully trains weak learners on the testing data set, fitting residual errors, and detecting its weak points. Based on such weak points, it generates a new weak learner ($\hbox {l}_i$) during every iteration. It evaluates its weight ($\omega _i$) in order to enhance the difference between the response value and the aggregated predicted value, hence increasing prediction accuracy. Finally, the algorithm updates the current model ($\hbox {M}_i$) by emphasising on the prior weak learner’s ($\hbox {M}_i$-1) weak point according to Eq. (3). It then integrates the weak learner into the existing model after training and iteratively generates a single strong learner ($\hbox {M}_n$, i.e., ensemble of weak learners).

3 $$\begin{aligned} M_{i} = M_{i-1} + \delta \cdot \omega _{i} \cdot l_{i} \quad (i=1,2,3,\ldots ,n) \end{aligned}$$

To explore further the predictor importance, we estimated the coefficients indicating the relative importance of each predictor within the trained model by computing the total variations in the node risk ($\Delta$*R*) due to split among each predictor, and then normalising it by the total number of branch nodes ($\hbox {R}_{{BN}}$) and is mathematically represented as:

4 $$\begin{aligned} \Delta R = \frac{R_{P}-(R_{CH1} + R_{CH2})}{R_{BN}} \end{aligned}$$

where $R_{P}$ indicates the node risk of the parent and $R_{CH1}$ & $R_{CH2}$ indicates the node risk of two children. The node risk at individual node (R$_{\hbox {i}}$) is mathematically represented as in Eq. (5);

5 $$\begin{aligned} R_{i} = P_{i} \cdot E_{i} \end{aligned}$$

where $P_{i}$ denotes the probability of node *i* and $E_{i}$ denotes the node *i* mean square error.

### Predictor sensitivity

We have performed the sensitivity analysis of the predictors using Partial Dependence Plot (PDP)^21,35^. PDP depicts whether a model’s predicted response (outcome) changes as a single explanatory variable varies. These plots have the advantage of exhibiting the form of relationship that exists between the variable and the response^36^. Moreover, it depicts the marginal effect of one or more variables on the predicted response of the model^37^. In this study, we have considered the combined impact of two predictors simultaneously from the input predictor set (i.e., $\upsilon$) on the predictand by marginalising the impact of the remaining predictors. To accomplish this, a subset $\upsilon ^{\hbox {s}}$ and a complimentary set ($\upsilon ^{\hbox {c}}$) of $\upsilon ^{\hbox {s}}$ is extracted from the predictor set ($\upsilon = \{z_{1}, z_{2},\ldots , z_{n}\}$) where n represents the total number of predictors. Any prediction on $\upsilon$ is determined by Eq. (6) and the partial dependence of the predictor in $\upsilon ^{\hbox {s}}$ is inferred by computing the expectation (*E*$_{c}$) of Eq. (6):

6 $$\begin{aligned}&f(\upsilon ) = f(\upsilon ^{s}, \upsilon ^{c}) \end{aligned}$$

7 $$\begin{aligned} \begin{aligned} f^{s}(\upsilon ^{s})&= E_{c}[f(\upsilon ^{s}, \upsilon ^{c})]\\&= \int f(\upsilon ^{s}, \upsilon ^{c}) \cdot \rho _{c}(\upsilon ^{c}) \cdot d\upsilon ^{c} \end{aligned} \end{aligned}$$

where $\rho _{c}(\upsilon ^{c}$) indicates the marginal probability of $\upsilon ^{c}$, which is represented in Eq. (8).

8 $$\begin{aligned} \rho _{c}(\upsilon ^{c}) \approx \int p(\upsilon ^{s}, \upsilon ^{c}) \cdot d\upsilon ^{s} \end{aligned}$$

Then, the partial dependency of the predictor in $\upsilon ^{s}$ can be determined by :

9 $$\begin{aligned} f^{s}(\upsilon ^{s}) \approx \frac{1}{U} \sum _{i=1}^{U} f(\upsilon ^{s}, \upsilon _{i}^{c}) \end{aligned}$$

where U represents the total number of observations.

### Automated machine learning model

AutoML is used to automate the machine learning process such as data pre-processing, predictor or feature engineering, best algorithm selection, and hyperparameter optimisation^38–40^. For past few years, it has been widely used in industry and academia to solve real and near real-time problems^41–43^. In this study, firstly, we have performed the predictor standardisation using Z-score scaling^44^. Afterward, we divided the complete dataset randomly using Mersenne Twister (MT) random generator in an 80:20 ratio for training and testing the AutoML model. The dimension of the complete dataset is 182 $\times$ 5, where 182 is the number of observations and 5 is the number of predictors (i.e., area of the region, sensing range of the sensor, transmission range of the sensor, and the number of sensors) and the response variable (i.e., *k*-barrier). The dimension of the training dataset is 145 $\times$ 5, and the dimension of the testing dataset is 37 $\times$ 5. After data division, we have automated the algorithms selection and hyperparameter optimisation step and investigated its performance. Various explainable machine learning models participate in the algorithm selection process, which is discussed next in the upcoming subsections.

#### Support vector regression model

The Support Vector Regression (SVR) model was introduced by Vapnik et al.^45^, and it was developed primarily using the Support Vector Machine (SVM) classifiers. The SVR model has the benefit of being able to optimise the nominal margin using regression task analysis and is a popular choice for prediction and curve-fitting both for linear and nonlinear regression types^46^. The relationship among input and output variables for nonlinear mapping^47^ is determined by:

10 $$\begin{aligned} y_{i} = w\phi (p) + q \end{aligned}$$

where *p*${ = (p^{1}, p^{2},\ldots , p^{n})}$ indicates the input, *y*$_{i}$ $\in$ Rl indicates the output, *w* $\in$ R$_{\hbox {n}}$ indicates the weight vector, *q* $\in$ R indicates the constant, *n* indicates the number of training datasets and $\phi (p)$ indicates an irregular function that is used to assign the input to the predictor. To determine *w* and *q*, Eq. (11) is used, where $\chi _{i}, \chi _{i}^{*}$ indicates the slack variable.

11 $$\begin{aligned} \begin{aligned} Minimise : \frac{1}{2}||w^{2}|| + C \sum _{i=1}^{n}(\chi _{i} - \chi _{i}^{*}) \\ Subject \; to : {\left\{ \begin{array}{ll} y_{i} - ( w\phi (p_{i}) + q_{i}) \le \epsilon + \chi _{i} \\ ( w\phi (p_{i}) + q_{i}) - y_{i} \le \epsilon + \chi _{i}^{*} \\ \chi _{i}, \chi _{i}^{*} \ge 0 \end{array}\right. } \end{aligned} \end{aligned}$$

In the SVR model, the three basic hyperparameters used are the insensitive loss function ($\epsilon$) that specifies the tolerance margin; the capacity parameter or penalty coefficient or box constraint (*C*) that specifies the error weight; and the Gaussian width parameter or kernel scale ($\gamma$)^48,49^. A high value of *C* lets SVR reminisce the training data. The smaller $\epsilon$ value implies noiseless data. However, the $\gamma$ value is equally responsible for the under-adjustment or over-adjustment of prediction. Mathematically, it is represented as:

12 $$\begin{aligned} K(p_{i},p) = e^{(-\gamma ||p_{i}-p||^{2})} \end{aligned}$$

where *K* represents the kernel function, $\gamma$ represents the kernel scale that manages the influence of predictors variation on kernel variation.

#### Gaussian process regression model

Gaussian Process Regression (GPR), also known as kriging^50^ is based on Bayesian theory^51^ and is used to solve complex regression problems (high dimension, nonlinearity), facilitates the hyper-parameter adaptive acquisition, easy to implement, and is used with no loss of performance. The fundamental and extensively used GPR is mainly comprised of a simple zero mean and squared exponential covariance function^52^ as represented in Eq. (13).

13 $$\begin{aligned} K(x,x\prime ) = \varpi _{f}^{2}exp \left[ \frac{-r}{2}\right] \end{aligned}$$

where

14 $$\begin{aligned} r = \frac{|x-x\prime |^{2}}{g^{2}} \end{aligned}$$

where $k(x,x\prime )$ represents the covariance function or kernels that provide the expected correlation among several observations. In the GPR model, there are two hyperparameters used, such as the model noise ($\varpi _{f}$) and the length scale (*g*) that regulates the vertical scale and the horizontal scale of the function change, respectively.

#### Binary decision tree regression

A Binary Decision Tree (BDT) regression is formed by performing consecutive recursive binary splits on variables, that is of the form *y*$_{i}$ $\le$ *v*, *y*$_{i}$ $\ge$ *v*, where *v* $\in$ ${\mathbb {R}}$ are observed values in a binary regression tree^53^, which is represented as:

15 $$\begin{aligned} T(y) = \sum _{m=1}^{M}m\; \cdot \; B_{m}(y) \end{aligned}$$

where *T(y)* indicates the regression tree, *M* indicates the number of tree’s terminal nodes, and *B*$_{m}$ *(y)* indicates the base function which is determined by:

16 $$\begin{aligned} B_{m}(y) = \prod _{i=1}^{L_{m}}\;[y_{i}(m) - v_{im}] \end{aligned}$$

where *L*$_{m}$ indicates the total splits, *y*$_{i}$ indicates the involved variable, and *v*$_{im}$ indicates the splitting value. Moreover, the decision tree establishes the rule till the samples in a leaf fall under a specified size, i.e., the minimum leaf (min-leaf) size^54^. Since the min-leaf size defines when splitting must be terminated, it is considered a vital parameter that must be fine-tuned.

#### Ensemble regression model

Perrone and Cooper^55^ proposed a general conceptual framework for obtaining considerably better regression estimates using ensemble methods. Ensemble Learning (EL) enhances performance by building and combining several base learners with specific approaches. It is mainly used when there is a limited amount of training data. It is challenging to choose a suitable classifier with this limited available data. Ensemble algorithms minimise the risk of selecting a poor classifier by averaging the votes of individual classifiers. This study has applied bagging and boosting EL methods due to their widespread usage and effectiveness for building ensemble learning algorithms.

Bagging (Breiman^56,57^), also known as bootstrap aggregation or Random Forest (RF), is one of the most prominent approach for building ensembles, that uses a bootstrap sampling technique to generate multiple different training sets. Subsequently, the base learners are trained on every training set, and then combining those base learners to create the final model. Hence, bagging works for a regression problem as follows: Consider a training set, *S* that comprises of data ${\{(X_{i},Y_{i}), i = 1,2,\ldots ,m\}}$, where *X*$_{i}$ and *Y*$_{i}$ represents the realisation of a multi-dimensional estimator and a real valued variable respectively. A predictor *P(Y|X = x) = f(x)*^58^ is represented as:

17 $$\begin{aligned} \zeta _{m}(x) = h_{m}(S_{1}, S_{2},\ldots S_{m})(x) \end{aligned}$$

At first, create a bootstrapped sample Eq. (18) based on the empirical distribution of the pairs *S*$_{i}$ = (*X*$_{i}$, *Y*$_{i}$), next, using the plug-in concept, estimate the bootstrapped predictor as shown in Eq. (19). Finally, the bagged estimator is represented by Eq. (20).

18 $$\begin{aligned} S_{i}^{*}= & {} (Y_{i}^{*},X_{i}^{*}) \end{aligned}$$

19 $$\begin{aligned} \zeta _{m}^{*}(x)= & {} h_{m}(S_{1}^{*}, S_{2}^{*},\ldots S_{m}^{*})(x) \end{aligned}$$

20 $$\begin{aligned} \zeta _{m;B}(x)= & {} P|S_{m}^{*}(x)| \end{aligned}$$

Moreover, the three hyperparameters used in bagging are the MinLeafSize (minimum number of observations per leaf), NumVariablesToSample (number of predictors to sample at every node), and the NumLearningCycles (number of trees). The first two parameters determine the tree’s structure, while tuning the final parameter helps balance efficiency and accuracy.

Boosting (Freund^59^) is another ensemble method that aims to boost the efficiency of a given learning algorithm. The Least-Squares Boosting (LSBoost) ensemble method is used in this study because it is suited for regression and forecasting problems. LSBoost aims to reduce the Mean Squared Error (MSE) between the target variable *(Y)* and the weak learners’ aggregated prediction (*Y*$_{p}$). At first, median of *(Y)*, represented as $({\widetilde{Y}}$) is computed. Next, to enhance the model accuracy, several regression trees (*r*$_{1}$, r$_{2}$,$\ldots$ , r$_{\hbox {m}}$) are integrated in a weighted manner. Individual regression trees are determined by the following predictor variables *(X)*^60^:

21 $$\begin{aligned} Y_{p}(X) = {\widetilde{Y}}(X) + \eta \sum _{m=1}^{d} w_{m} \times r_{m}(X) \end{aligned}$$

where *(w*$_{m}$) represents the weight for the *m* model, *d* represents the weak learners, and $\eta$ with $0 < \eta \le$ 1 represents the learning rate.

#### Kernel regression model

Kernel regression (Nadaraya^61^) is the most used non-parametric method on account of the virtue of kernel and is undoubtedly known as univariate kernel smoother. In order to achieve a kernel regression, a collection of kernels are locally placed at every observational point. The kernel is set a weight to every location depending on its distance from the observational point. A multivariate kernel regression^62^ determines how the response parameter, *y*$_{i}$ is dependent on the explanatory parameter, *x*$_{i}$, as in Eqs. (22) and (23).

22 $$\begin{aligned} E(y_{i}|x_{i}) = m(x_{i}) + \psi _{i} \end{aligned}$$

and

23 $$\begin{aligned} y_{i} = m(x_{i}) + \psi _{i} \end{aligned}$$

where $E[\psi _{i}] = Cov[m(x_{i}),\psi _{i}] = 0$, *m*(.) represents a non-linear function, and $\psi _{i}$ is random with mean zero and variance $\sigma ^{2}$. It describes the way that *y*$_{i}$ varies around its mean, *m(x*$_{i}$). The mean can be represented as the probability density function *f*:

24 $$\begin{aligned} m(x_{i}) = E[Y_{i}|x_{i}=x] = \frac{\int y . f(x,y)dy}{\int f(x,y)dy} = \frac{\int y . f(x,y)dy}{\int f(x)} \end{aligned}$$

#### Linear regression model

A linear regression model^63^ examines the relationship among different influential predictors and an outcome variable. The basic linear regression model, which represents the universal set of two-variable and multiple regression as complementary subsets, can be expressed as:

25 $$\begin{aligned} Y = a + \sum _{i=1}^{n} b_{i}X_{i} + u \end{aligned}$$

where *Y* represents the dependent variable, $X_{1}, X_{2},\ldots , X_{n}$ represents the *n* independent variables, *a* and *b* represents the regression coefficients and *u* represents the stochastic disturbance-term that could be caused by an undefined independent variable.

#### Bayesian optimisation

Bayesian Optimisation (BO)^64,65^ is an efficient approach for addressing optimisation problems characterised by expensive experiments. It keeps track of the previous observations and forms a probabilistic mapping (or model) between the hyperparameter and a probabilistic score on the objective function that is to be optimised. The probabilistic model is known as a surrogate of the objective function. The surrogate function is much easy to optimise, and with the help of the acquisition function, the next set of hyperparameters is selected for evaluation on the actual objective function based on its best performance on the surrogate function. Hence, it comprises a surrogate function for determining the objective function and an acquisition function for sampling the next observation. In BO, the objective function *(f)* is obtained from the Gaussian Process (GP) as described in Eq. (26).

26 $$\begin{aligned} f(x) \sim GP (\mu (x), \vartheta (x_{i},x_{j})) \end{aligned}$$

where $\mu$ and $\vartheta$ are calculated from the observations of *x*^66^.

**Figure 1:**
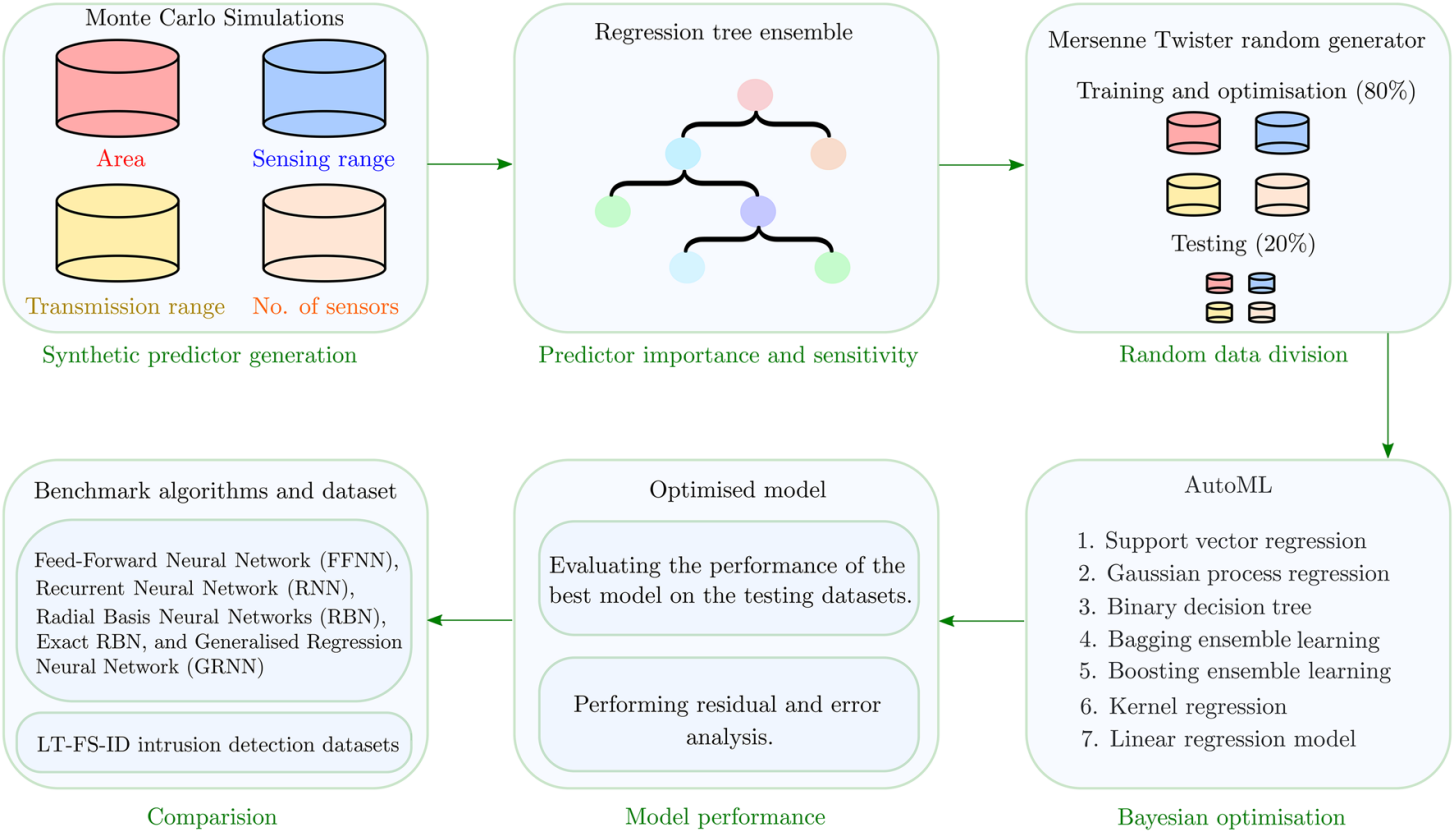
Flowchart of the proposed methodology.

We select the best performing algorithm among the above-discussed models with the optimised hyperparameter. Lastly, we evaluated the performance of the best-performing algorithm using the test dataset. A flowchart of the detailed methodology is illustrated in Fig. 1.

**Figure 2:**
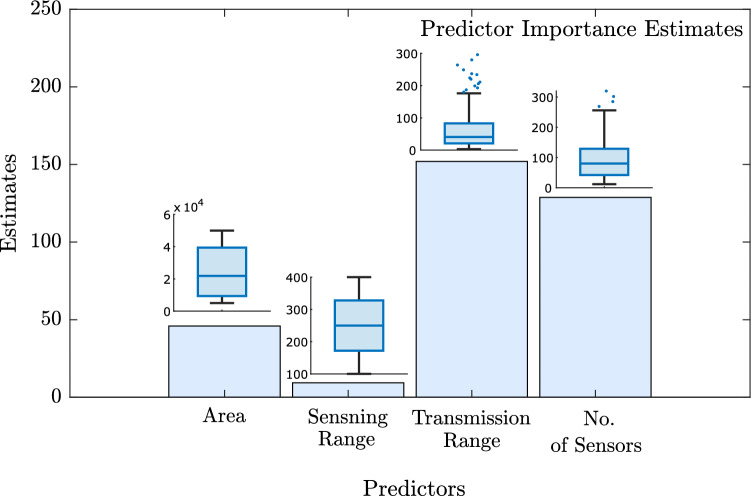
Graph showing the relative predictor importance score for all four predictors. The estimates for the area of the RoI, sensing range of the sensor, transmission range of the sensor, and the number of sensors are 46.0, 9.3, 152.0, and 128.9, respectively.

## Results

### Predictor importance and sensitivity

We plotted the relative predictor importance score of each predictor along with their respective box plot for a better visual representation of the datasets (Fig. 2). We found that the relative predictor importance score ranges approximately from 9 to 152. The higher the value of the relative estimate, the more relevant is the predictor in estimating the response variable (i.e., *k*-barriers). We found that out of these four predictors, the transmission range of the sensor emerges as the most relevant predictor in predicting the required number of *k*-barriers for fast intrusion detection and prevention considering Gaussian node distribution over a rectangular region. The number of sensors also shows good relevancy in predicting the response variable and ranked second. The area of the region of interest and the sensing range of the sensor shows fair relevancy and ranked third and fourth, respectively.

**Figure 3:**
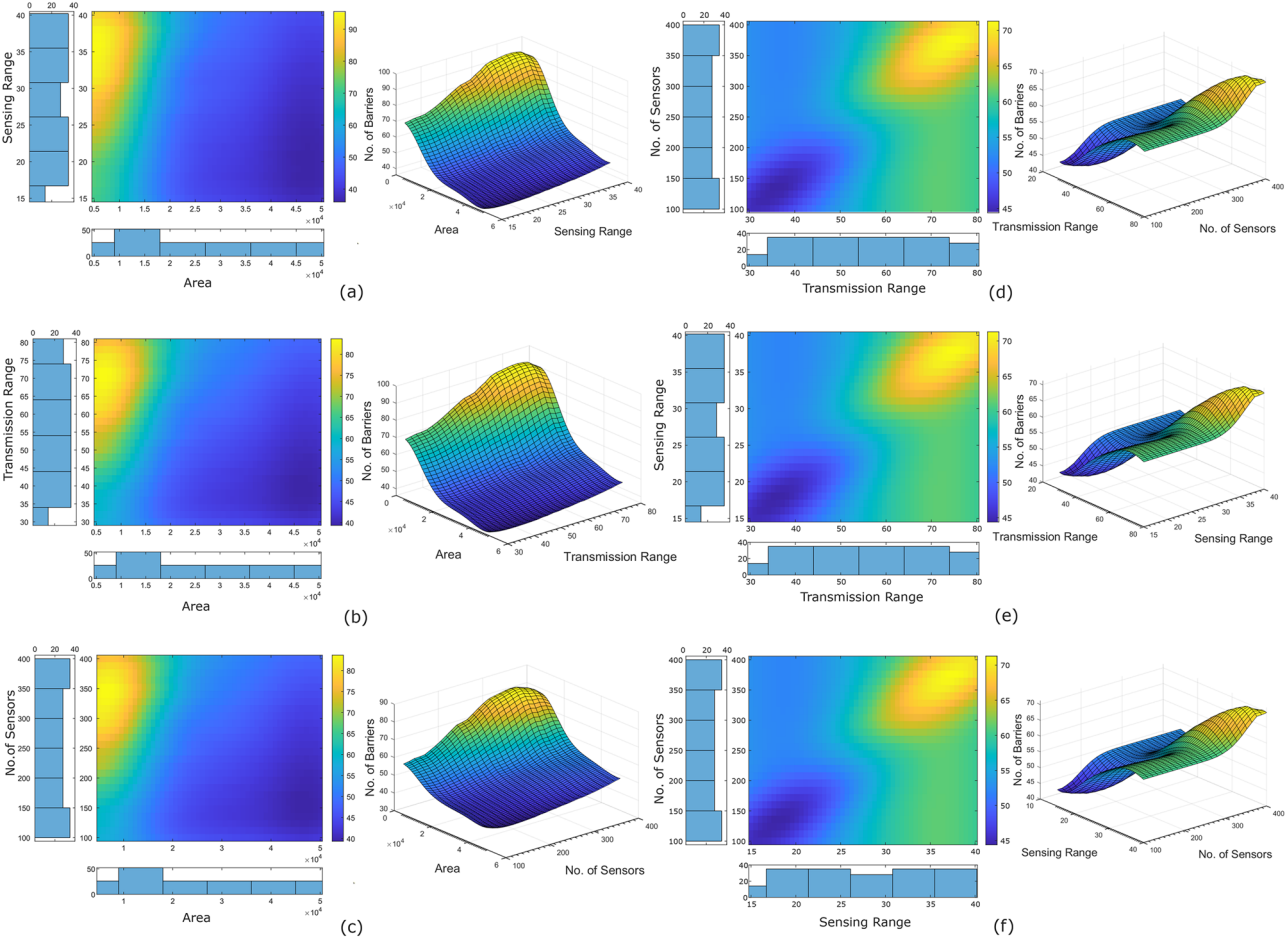
Two-dimensional and three-dimensional partial dependency plots show the predictor sensitivity of all possible predictor pairs. The histogram along the x and y-axis of the two-dimensional plot shows the distribution of the predictor and the response variable, respectively.

We also evaluated the impact of each predictor on the response variable. We plotted the partial dependence plot for each possible pair of predictors (Fig. 3a–f). For a better visual inspection, we also plotted the three-dimensional plot and its two-dimensional illustration. We observed that the area of the RoI has a slightly negative impact on the target variable i.e., the response variable decreases with an increase in the area of the RoI. However, an inverse relationship is observed with all other predictors. The sensing range of the sensor, the transmission range of the sensor, and the number of the sensors have a positive impact on the response variable i.e., the response variable increases with an increase in these predictors.

### Model performance

**Figure 4:**
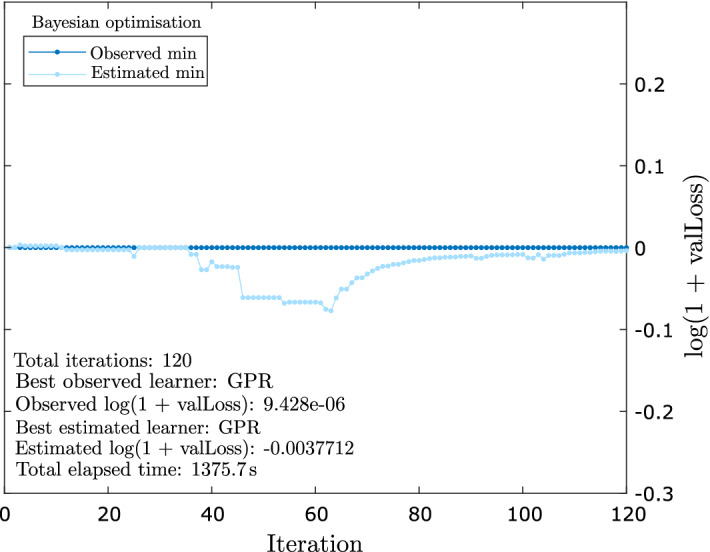
Curve illustrating the Bayesian optimisation process for the selection of the best machine learning model with optimal hyperparameters.

We iteratively selected the best machine learning model with optimised hyperparameters value using the Bayesian optimisation^67–69^ on the 80% of the datasets (Fig. 4). We used Eq. (27) as the objective function (*Obj*) to select the best machine learning model with optimised hyperparameters.

27 $$\begin{aligned} Obj = log(1+valLoss) \end{aligned}$$

where *valLoss* is the cross-validation mean square error (CV-MSE). At each iteration, the value of the objective function is computed for any one of the participating models. The model (with optimised hyperparameters), which returns the minimum observed loss (i.e., the smallest value of the objective function so far), is considered as the best model. After iterating for 120 iterations, the AutoML algorithm returned the GPR model as the best model along with the optimal hyperparameters (i.e., for the GPR model; sigma = $\mathrm {0.98}$). Before returning the model, the AutoML algorithm retrains the GPR model on the entire training dataset.

**Figure 5:**
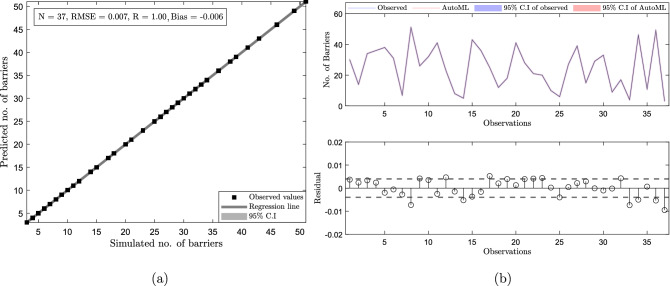
The left panel shows the linear regression plot between the predicted and observed responses. The top plot on the right panel shows the time series plot of the predicted and observed. The bottom panel shows the corresponding residuals. The dashed line in the residual plot shows the RMSE value.

Once we get the trained GPR model, we evaluate its performance on the training datasets to estimate the training accuracy. We found that the model performed well on the training datasets with a correlation coefficient (R = 1), root mean square error (RMSE = 0.003), and bias = 0. However, for an unbiased evaluation, we evaluated the performance of the trained model on the test datasets (i.e., 20% of the total datasets). In doing so, we fed the testing predictors into the trained GPR model and obtained the predicted response. We then compared the GPR predicted *k*-barriers with the observed values (Fig. 5a). We found that the GRP model performs prodigiously with a R = 1, RMSE = 0.007, and bias = − 0.006. All the data points are aligned along the regression line and lie well inside the 95% Confidence Interval (C.I).

Further, to assess the appropriateness of the plotted linear regression plot, we performed residual analysis. We plotted the time series of the observed and the predicted values along with the corresponding residual values (Fig. 5b). We found that the residuals are significantly low and do not follow any pattern, which indicates a good linear fit.

**Figure 6:**
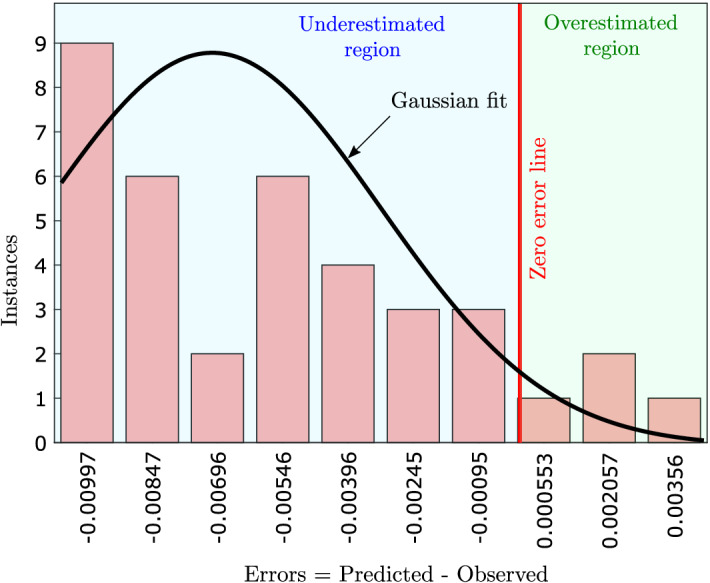
Error analysis using error histogram of 10 bins. The line in red shows the zero error line. The area to the left of the zero error line shows the underestimated region, and the area right to the zero error line shows the overestimated region.

To understand the distribution of the error (i.e., difference of predicted and observed values), we performed error analysis using error histogram (Fig. 6). To do so, we plotted the error histogram using ten bins. The error ranges from $\mathrm {-0.00997}$ from the left to $\mathrm {0.00356}$ on the right of the histogram plot. We found that the error follows a right-skewed Gaussian distribution. The peak of the distribution lies in the underestimated region. Lastly, we presented the results of the remaining algorithms of the AutoML (i.e., SVR, BDT, Bagging ensemble learning, Boosting ensemble learning, kernel, and linear regression) in Table 2. We found that the best performing AutoML algorithm (i.e., GPR) outperforms all the other algorithms.

**Table 2:** Performance of the other AutoML algorithms.

Performance metrics	Algorithms					
	SVR	BDT	Bagging EL (random forest)	Boosting EL (LSBoost)	Kernel regression	Linear regression
R	0.93	0.81	0.93	0.73	0.91	0.94
RMSE	63.61	73.07	81.84	118.03	32.29	33.68
Bias	53.59	56.99	67.31	89.81	31.28	31.81
t (s)	95.3	111.3	103.4	107.7	43.01	36.7

## Discussion

We observed that the AutoML approach successfully selects the best machine learning model among a group of explainable machine learning algorithms (i.e., among SVR, GPR, BDT, bagging ensemble learning, boosting ensemble learning, kernel regression, and linear regression model) and optimised its hyperparameters. However, we have compared the AutoML derived results with the benchmark algorithms for an unbiased and fair evaluation of the proposed approach. We selected Feed-Forward Neural Network (FFNN)^70^, Recurrent Neural Network (RNN)^71^, Radial Basis Neural Networks (RBN)^72^, Exact RBN^73^, and Generalised Regression Neural Network (GRNN)^74^ as the benchmark algorithms. We selected these algorithms because they are frequently used in diverse applications such as remote sensing, blockchain, cancer diagnosis, precision medicine, decease prediction, self-driving cars, streamflow forecasting, and speech recognition; hence have high generalisation capabilities^37,75–77^. In doing so, we trained these algorithms over the same datasets. We found that the AutoML outperforms all the deep learning benchmark algorithms (Table 3). Among the benchmark algorithms GRNN performs the best (with R = 0.97, RMSE = 64.61, Bias = 60.18, and computational time complexity, t = 2.23 s). Surprisingly, all the benchmark algorithms have a high positive bias value. It indicates that these models highly overestimate the number of required *k*-barriers. We have also compared the performance of the AutoML with previous studies^21,22^ for the prediction of *k*-barriers and *k*-barrier coverage probability (Table 4).

**Table 3:** Comparing the performance of the AutoML with the deep learning models.

Performance metrics	FFNN	RNN	Exact RBN	RBN	GRNN
R	0.47	0.95	0.30	0.41	0.97
RMSE	36.96	14.92	107.95	161.11	64.61
Bias	21.47	71.06	86.21	139.23	60.18
t (s)	2.5	13.51	2.90	3.98	2.23

Further, we also tested the performance of the AutoML approach over the publicly available intrusion detection dataset^22^. In a recent study, Singh et al.^22^ have proposed a log-transformed feature scaling based algorithm (i.e., LT-FS-ID) for intrusion detection considering uniform node distribution scenario. We downloaded the datasets and applied the proposed AutoML approach to them. In doing so, we iterated the AutoML for 120 iterations using the Bayesian optimisation to obtain the best optimised machine learning model. We found that AutoML approach perform well over the dataset (with R = 0.92, RMSE = 30.59, and Bias = 18.13). Interestingly, the same GPR algorithms emerges as the best learner algorithms with a optimised sigma = 0.33. It highlights the potential of the GPR algorithm for intrusion detection, which becomes more apparent from the recently published literature’s^21,78^.

The proposed AutoML approach for estimating the *k*-barriers for fast intrusion detection and prevention is highly user-friendly and provides a fast solution. It reduces the confusion of selecting the best-performing algorithm by automating the process. Further, it also overcomes the limitation of the LT-FS-ID algorithm^22^. LT-FS-ID algorithm only works if the input predictors are a positive real number. It will not work if any input predictors contain zero (or negative values). Although the AutoML approach gives the best result, its performance will hamper with the sensor aging. In other words, with the aging effect in the sensors, the quality of the data recorded by the sensor may change drastically (i.e., datasets become dynamic), resulting in performance degradation. In such a situation, retraining the proposed model will solve the problem.

**Table 4:** Comparing the results of AutoML with previous studies.

Performance metrics	*k*-barriers		*k*-barrier coverage probability^21^		
	AutoML (This study)	LT-FS-ID^22^	GPR	S-GPR	C-GPR
R	1	0.98	0.85	0.64	0.79
RMSE	0.007	6.47	0.095	0.137	0.108
t (s)	0.73	0.65	8.16	7.79	9.51

## Conclusion

In this study, we proposed a robust AutoML approach to estimate the accurate number of *k*-barriers required for fast intrusion detection and prevention using WSNs over a rectangular RoI considering the Gaussian distribution of the node deployment. We found that the synthetic predictors (i.e., the area of the RoI, sensing range of the sensor node, transmission range of the sensor node, and the number of sensors) extracted through Monte Carlo simulations successfully mapped with the *k*-barriers. Among these predictors, the transmission range of the sensor emerges as the most relevant predictor, and the sensing range of the sensor emerges as the least relevant predictor. In addition to this, we observed that only the area of the RoI has a slightly negative impact on the response variable. We then iteratively run the AutoML algorithms to obtain the best machine learning model among the explainable machine learning model using Bayesian optimisation techniques. We found that the AutoML algorithm selects the GPR algorithm as the best machine learning model to map the required *k*-barriers accurately. We evaluated the potential of the GPR algorithm over unseen test datasets. We found that the AutoML elected algorithm performs exceptionally well on the test datasets.

We further compared the AutoML results with the benchmark algorithms for a more reliable and robust conclusion. We found that AutoML outperforms all the benchmark algorithms in terms of accuracy. For more generalisation of this approach, we tested the efficacy of the AutoML over the publicly available datasets on intrusion detection using WSNs, and we found a similar performance. This study is a step towards a cost-efficient approach for fast intrusion detection and prevention using explainable machine learning models.

## Data Availability

The datasets generated during and/or analysed during the current study can be made available from the corresponding author on a reasonable request.

## Code availability

The computer algorithms originated during the current study can be made available from the corresponding author on a reasonable request.
